# Falls in community-dwelling women with bipolar disorder: a case–control study

**DOI:** 10.1186/s12888-022-04258-7

**Published:** 2022-09-20

**Authors:** Amanda L. Stuart, Julie A. Pasco, Michael Berk, Shae E. Quirk, Heli Koivumaa-Honkanen, Risto Honkanen, Mohammadreza Mohebbi, Lana J. Williams

**Affiliations:** 1 School of Medicine, IMPACT the Institute for Mental and Physical Health and Clinical Translation, Deakin University, Barwon Health, PO Box 281, Geelong, 3220 Australia; 2 Barwon Health University Hospital, Geelong, Australia; 3 Department of Medicine-Western Health, The University of Melbourne, St Albans, Australia; 4 Department of Epidemiology and Preventive Medicine, Monash University, Melbourne, Australia; 5 Department of Psychiatry, The University of Melbourne, Parkville, Australia; 6 Florey Institute of Neuroscience and Mental Health, Parkville, Australia; 7 Orygen the National Centre of Excellence in Youth Mental Health, Parkville, Australia; 8 Institute of Clinical Medicine/Psychiatry, University of Eastern Finland, Kuopio, Finland; 9 Institute of Clinical Medicine, Kuopio Musculoskeletal Research Unit (KMRU), University of Eastern Finland, Kuopio, Finland; 10 Mental Health and Wellbeing Center, Kuopio University Hospital, Kuopio, Finland; 11 Faculty of Health, Deakin University, Burwood, Australia

**Keywords:** Bipolar disorder, Fall, Psychotropic medication, Case–control, Psychiatry, Neuroscience, Mental disorders, Mania, Depression

## Abstract

**Background:**

Falls are a common occurrence in psychiatric hospital settings, however population-based research among individuals with psychiatric disorders, in particular bipolar disorder (BD) is scant. Thus, we aimed to investigate falls risk in community-dwelling women diagnosed with BD.

**Methods:**

Women with BD (cases, *n* = 119) were recruited from health care settings located in southeast Victoria, Australia. Age-matched controls (*n* = 357, ratio 3:1) without BD were participants in the Geelong Osteoporosis Study drawn from the same geographical region. Lifetime history of BD was identified by semi-structured clinical interview (SCID-IV/NP). Previous 12-month falls data were obtained via questionnaire. Information on mobility, alcohol use, general health, medication use, blood pressure, body mass index, socioeconomic status and use of a walking aid was collected. Generalised Estimating Equations, binary and ordinal logistic regression were used to determine the odds ratio (OR) and 95% confidence interval (CI) for falls following adjustment for confounders.

**Results:**

During the 12-month period, 34 (28.6%, median age 48.4 yr) cases and 70 (19.6%, median age 49.1 yr) controls reported one fall; 22 (18.5%) cases and 18 (5.0%) controls reported ≥ two falls (*p* < 0.001). Cases had 2.5-fold increased odds of at least one fall and 2.9-fold increased likelihood of increasing falls categories (0 vs. 1 vs. 2 +), compared to controls [adjOR 2.5, 95%CI (1.8, 3.4), adjOR OR 2.9, 95%CI (2.0, 4.1)].

**Conclusion:**

Risk of falls was greater among women with BD. Balance training could be a research and clinical focus for falls prevention programs among women with bipolar disorder to prevent the detrimental outcomes associated with falling.

## Introduction

Bipolar spectrum disorders are characterised by recurrent, chronic, and significant fluctuations in emotionality and energy, estimated to affect approximately 1.2% of adults worldwide [1, 2]. Bipolar disorder is highly comorbid with both psychiatric and medical conditions in turn increasing the associated disease burden [3]. Identification of co-occurring conditions can help detect and prevent potential adverse outcomes for patients.

Globally, falls are a leading cause of severe, unintentional injury including bone fracture and brain or internal injury often causing fatal outcomes. Falls are also commonly associated with minor injuries, such as wounds, contusions and strains [4]. Lower quality of life [5] and subsequent fear of falling [6] are also adverse consequences of falling. Falls can cause a heavy burden to the healthcare system; falls are responsible for approximately 40% of hospitalisations due to injury in Australia (2019–20 data), and the cost of treating a fall accounted for over $4 billion healthcare expenditure (2018–19 data) [7, 8].

Falling has been reported to be more common among the elderly and women [9]. Furthermore, we have previously reported that unipolar depression, anxiety disorders and psychotropic medication used in its treatment are independently associated with an increased risk of falls in men and women spanning the full adult age range (20–93 years) [10–12]. A few systematic reviews have now been published supporting these findings, albeit in elderly samples [13–16].

Evidence is starting to emerge suggesting falls are also common among those with a diagnosis of bipolar disorder. In a psychiatric inpatient setting, Tseng and colleagues [17] identified bipolar disorder as a risk factor for falling once (OR 8.55, 95%CI 5.66–12.93), as well as for falling multiple times (> 3 falls OR 14.14, 5.65–35.40), compared to inpatients with neurosis. Lavsa et al. [18] also reported that fallers were more likely to be diagnosed with bipolar disorder than non-fallers when comparing 774 fallers with age-, sex- and admission year-matched non-falling inpatients at a psychiatric medical centre (20.8% vs. 14.5%, *p* = 0.001). In addition, Hendrie and colleagues [19] reported a 2-fold higher likelihood of experiencing falls for a group of 255 older patients with BD and other serious mental illnesses (OR 2.16, 95%CI 1.52–3.08), compared to a group of primary care patients. Whether or not these relationships still exist among those residing in the population independent of known risk factors, such as the use of a walking aid, blood pressure, socioeconomic status, mobility and alcohol intake is unknown [20–22].

Given the paucity of published data especially spanning the full adult population, we investigated falls risk in a community-dwelling sample of women diagnosed with bipolar disorder, adjusting for known potential confounders.

## Method

### Participants

Women with bipolar disorder (*n* = 119, median age 48.4 yr, range 21-79 yr) were recruited from public and private healthcare settings (2011–2018) from the Barwon Statistical Division (BSD), Victoria, Australia. To be eligible, participants were required to give informed consent, be aged > 20 years, and meet criteria for bipolar disorder I, II or not otherwise specified (NOS) using the Structured Clinical Interview for DSM-IV-TR Research Version, Non-patient edition (SCID-I/NP) [23]. SCID-I/NP interviews were conducted by trained personnel with post-graduate qualifications in psychology under the supervision of a psychiatrist. For inclusion in the current analyses, the diagnosis of bipolar disorder preceded the 12-month falls ascertainment period (see the following section below for further details regarding falls assessment). A full description of the study design has been published [24].

Controls without bipolar disorder (confirmed using SCID-I/NP) were drawn from the Geelong Osteoporosis Study (GOS), a population-based cohort randomly-selected from the Australian electoral rolls [25]. Originally, 1494 women were recruited from the BSD between 1993 and 1997, returning for a health assessment at two-to-five-year intervals. A further 246 women were recruited between 2006 and 2008 to replenish the 20–29-year age group. Of the 1126 women who completed the 10-year GOS assessment phase (2004–2008), 926 had complete falls and psychiatric data with no history of bipolar disorder (determined as per the cases above). From this pool of 926 participants, controls were age-matched in 10-year age bands at a frequency of three to one using random ID selection. Thus, 357 controls (median age 49.1 yr, range 21-75 yr) were included in the current analyses.

Written informed consent was obtained from all participants and the Barwon Health Human Research Ethics Committee approved the research program (reference numbers 10/89 and 92/01). This research was carried out in accordance with ethical standards of the Declaration of Helsinki and later amendments.

### Assessments

Falls data were self-reported. A fall was defined as “when you suddenly find yourself on the ground, without intending to get there, after you were in either a lying, sitting or standing position” [26]. Participants were asked to provide details of up to three falls that occurred during the 12-month period prior to their assessment, including a description of the fall.

Current medication use was self-reported. Antidepressants, antipsychotics, sedatives/hypnotics and both short- and long-acting benzodiazepines were identified, as these have been previously implicated in falls risk [15, 16].

Weight was measured to the nearest 0.1 kg using electronic scales and height to the nearest 0.1 cm using a wall-mounted Harpenden stadiometer to calculate body mass index (BMI, kg/m^2^). Seated blood pressure was measured using a digital meter (A&D Company, model UA-751). Use of a walking aid (yes/no), general health (excellent/very good vs. good vs. fair/ poor) and mobility were self-reported. Mobility was categorised as ‘very active’ if participants engaged in vigorous activity regularly, ‘active’ if participants engaged in light activity regularly and ‘sedentary’ if they performed normal daily activities but no appreciable exercise, and/or were only able to perform light housework or equivalent. Alcohol intake (g/day) was estimated using the Cancer Council of Victoria Dietary Questionnaire for Epidemiological Studies [27].

Area-based socioeconomic status (SES) was determined using the Australian Bureau of Statistics Census data and the socio-economic indexes for areas (SEIFA). The SEIFA index of relative socioeconomic advantage and disadvantage (IRSAD) indicates the relative socioeconomic status of local areas based on the census data such as employment, income and education levels, occupation and housing data. IRSAD data were presented in quintiles, where the lowest quintile indicated the most disadvantaged group and the highest quintile indicated the most advantaged group [28].

### Statistical analyses

Comparisons of participant’ characteristics between those with a history of bipolar disorder (cases) compared to those with no history of bipolar disorder (controls) were undertaken using bivariate Generalised Estimating Equations (GEE) models and reported in Table 1. The implemented GEE models account for matching structure of the study design in the comparison of cases and controls.

**Table 1:** Characteristics for cases and controls. Data are presented as median (IQR), mean ± standard deviation, or n (%)

	Cases *n* = 119	Controls *n* = 357	*P* value
Age (yr)	48.4 (39.2–57.5)	49.1 (38.7–58.2)	0.887
BMI (kg/m^2^)	28.0 (24.4–34.0)	27.0 (23.6–32.4)	0.047
Diastolic BP (mmHg)	79 ± 11	77 ± 11	< 0.001
Systolic BP (mmHg)	129 ± 17	124 ± 16	< 0.001
Alcohol consumption (g/day)	3.0 (0.4–11.4)	3.8 (0.5–12.9)	0.595
Walking aid (current)	3 (2.6)	8 (2.3)	0.834
General health status (current)			
Excellent	47 (39.5)	208 (58.4)	< 0.001
Good	39 (32.8)	109 (30.6)	
Poor	33 (27.7)	39 (11.0)	
Mobility (current)			
Very active	25 (21.4)	97 (27.3)	< 0.001
Active	59 (50.4)	205 (57.6)	
Sedentary	33 (28.2)	54 (15.2)	
Socio-economic status (current)			
Quintile 1 (most disadvantaged)	14 (15.2)	54 (15.2)	0.506
Quintile 2	15 (16.3)	75 (21.1)	
Quintile 3	32 (34.8)	80 (22.5)	
Quintile 4	20 (21.7)	70 (19.7)	
Quintile 5 (most advantaged)	11 (12.0)	76 (21.4)	
Psychotropic medication use (current)			
Antidepressants	58 (48.7)	51 (14.3)	< 0.001
Sedatives/hypnotics	6 (5.0)	10 (2.8)	0.051
Benzodiazepines	4 (3.4)	15 (4.2)	0.367
Antipsychotics	78 (65.6)	1 (0.28)	< 0.001
Falls			
No falls	63 (52.9)	269 (75.4)	< 0.001
One fall	34 (28.6)	70 (19.6)	
Two or more falls	22 (18.5)	18 (5.0)	

Missing values- BMI (body mass index) *n* = 15, BP (blood pressure) *n* = 34, alcohol consumption *n* = 26, walking aid *n* = 4, health status *n* = 1, mobility *n* = 3, socio-economic status *n* = 29

A binary logistic GEE model was used to estimate the odds ratio (OR) and 95% confidence intervals (CI) for the likelihood of at least one fall among those with bipolar disorder. In the second analyses, falls were considered as an ordinal response; no falls, one fall and 2 + falls. An ordinal logistic regression using GEE technique was used to estimate the ordinal odds of falls among cases compared to controls. Potential confounding variables were tested and retained in the final models if *p* < 0.1 using a backwards stepwise regression technique. We presented both the unadjusted and final models. Two-way interactions were tested in the final models. Statistical analyses were performed using Minitab (version 18; Minitab, State College, PA) and IBM SPSS Statistics for Windows (version 27; IBM Corp, Armonk, NY).

## Results

Table 1 presents characteristics of the cases and controls. Cases had higher BMI and blood pressure and were less active than controls. They were also more likely to take sedatives/hypnotics, antidepressants and antipsychotics, and to report poor general health and falls in the past 12-months, compared to controls. The mean duration between diagnosis of bipolar disorder and the study assessment date for the cases was 24.9 years (± 13.2).

A total of 198 falls were documented during the 12-month period prior to assessment: 87 falls among 56 cases and 111 falls among 88 controls. Common causes of falls among cases were slippery surfaces (14.5% of falls), uneven surfaces (14.5%), stairs (13.2%) and falls due to feeling faint/dizzy (13.2%), whereas the common causes of falls for the controls were tripping over an obstacle (31.3%), slippery surfaces (21.9%), stairs (12.5%) and uneven surfaces (12.5%).

Cases were at 2.7-fold increased odds of reporting at least one fall during the past 12-months [unadjusted OR 2.7, 95%CI (2.1, 3.7), *p* < 0.001]. Following backwards elimination of non-significant variables, the final model was adjusted for age, blood pressure, alcohol consumption, benzodiazepine use and health status; the odds of falling at least once during the past 12-months were increased 2.5-fold [adjusted OR 2.5, 95%CI (1.7, 3.4), *p* < 0.001, Table 2].

**Table 2:** Logistic regression model (left panel) and ordinal logistic regression model (right panel) for the association between bipolar disorder and (i) risk of falling at least once and (ii) risk of ordinal increase in falls category. Results are displayed as odds ratio and 95% confidence intervals

	**At least one fall**				**Ordinal falls category (0 vs. 1 or 2 + falls)**			
	**Unadjusted model**		**Final model**		**Unadjusted model**		**Final model**	
	**OR (95%CI)**	***p***	**OR (95%CI)**	***p***	**OR (95%CI)**	***p***	**OR (95%CI)**	***p***
Bipolar disorder	2.71 (0.24,0.46)	< 0.001	2.46 (1.78,3.40)	< 0.001	2.92 (2.12,4.02)	< 0.001	2.85 (2.00,4.06)	< 0.001
Age	-	-	1.02 (1.00,1.04)	0.035			1.02 (1.00,1.04)	0.023
Systolic blood pressure	-	-	0.99 (0.98,0.99)	0.038			0.99 (0.98,1.00)	0.070
Diastolic blood pressure	-	-	1.01 (1.00,1.02)	0.016			1.01 (1.00,1.03)	< 0.001
Alcohol consumption	-	-	1.01 (1.00,1.02)	0.004			1.01 (1.00,1.02)	0.010
Benzodiazepine use	-	-	2.97 (0.94,9.43)	0.064			3.51 (0.92,13.4)	0.067
Health status – excellent/very good	-	-	-	-			-	-
Health status – good	-	-	1.30 (0.96,1.75)	0.089			-	-
Health status – fair/poor	-	-	1.38 (1.04,1.84)	0.028			-	-

*Note*- Generalised estimated equation model to account for the matching nature of the data

Cases had an almost 3-fold increased likelihood to be in each of the increasing fall categories (0 vs. 1 or 2 + falls), compared to controls [ordinal OR for being in one level higher fall category 2.9, 95%CI (2.1, 4.0), *p* < 0.001]. This increased ordinal odds remained significant following adjustment for the age, blood pressure, alcohol consumption and benzodiazepine use [adjusted ordinal OR for being in one level higher fall category 2.9, 95%CI (2.0, 4.1), *p* < 0.001, Table 2].

## Discussion

This population-based, case–control study demonstrates a two-fold risk of women with bipolar disorder having at least one fall and a three-fold increased likelihood of two or more falls over a 12-month period. This relationship was independent of known risk factors for falls, including age, blood pressure, alcohol consumption, benzodiazepine use and health status.

The results of the present study align with three prior studies conducted in patients with bipolar disorders residing in inpatient and clinical settings [17–19], of which, all reported a higher risk of falls among people diagnosed with bipolar disorder compared to those without bipolar disorder. Our results are of a smaller magnitude than Tseng et al. [17] who reported an 8-fold increased likelihood of one fall and a 14-fold increased likelihood of > 3 falls for inpatients with bipolar disorder, compared to inpatients with neurosis. This difference could be attributed to the severity of illness or higher medication doses taken during hospitalisations. Direct comparisons between our study and like studies were not possible given the paucity of data surrounding falls in community-dwelling women with bipolar disorder.

Several factors are likely to be at play in the association between bipolar disorder and increased falls. Psychotropic medication use has been implicated as a contributor to increased falls among psychiatric inpatient populations [15, 16, 18]. Patients taking medications for the treatment of bipolar disorder can be subject to a range of gait-related disturbances; side effects listed for antipsychotic use include parkinsonism, tardive dyskinesia and akathisia [29] and anticonvulsant and benzodiazepine use has been associated with ataxia, gait unsteadiness and balance issues [30, 31]. Furthermore, the impact of these medications on recall of falls is unknown. In the present study we tested psychotropic medication use in each statistical model and found benzodiazepine use to be the only significant confounder.

Gait and balance disturbances may also be a mechanistic link between mood disorders more broadly and falls. For example, increased gait speed and force exertion has been reported for patients with bipolar disorder experiencing a manic episode [32], impaired balance control during dynamic movements for patients experiencing a depressive episode [33] and increased postural sway in euthymic patients [34]. Another study reported a difference in swing time variability, a measure of unsteady gait, among those with bipolar disorder (phase unknown), suggesting these gait changes may predispose those with mood disorders to falls [35]. Thus, it seems that gait and balance disturbances should be targeted in falls research to help explore the excess risk of falls among women with bipolar disorder [36].

Furthermore, balance training could be a potential focus for falls prevention programs among women with bipolar disorder. Existing fall prevention programs, usually targeted to the elderly, vary in the type of exercises included, however gait, balance, and functional training are found to be the most effective with long-term falls prevention benefits [37]. A review of the literature on fall prevention programs targeting psychiatric inpatients identified only three intervention studies, with one including ambulating exercises and physiotherapist and occupational therapist involvement. Medication profiling and blood pressure monitoring were thought to be significant in preventing falls among inpatients [38]. A study investigating outcomes in a fall prevention program among depressed older adults found that improvement in depressive symptoms was associated with improvement in falls efficacy, suggesting interventions should target both mental health and fall prevention simultaneously [39]. Whether this would also be efficacious in bipolar disorder is unknown and could be a target for future research.

Strengths and limitations of the current study must be considered when interpreting the results. Strengths include use of the SCID-I/NP when identifying bipolar disorder, and the ability to compare bipolar disorder to age-matched controls drawn from the same geographical area. Previous falls research among women with a diagnosis of bipolar disorder has been completed in hospital settings, which generally reflects patients in an acute phase of illness, potentially with higher doses of medications; participants of the current study were community-dwelling women. Although participants were well at the time of appointment, there is a possibility that falls may have occurred during a depressive or manic episode or in a hospital/clinical setting, which was not documented as part of the falls questionnaire. The use of a 12-month recall for identification of falls is a further limitation, however, previous research has reported reasonable sensitivity between 12-month recall and a daily falls calendar, albeit in elderly samples [40, 41]. Furthermore, cognition was not measured in the current study which could have impacted recall; fallers with poor cognition are less likely to recall falling [41]. The 12-month time periods for which falls were recorded differed between the cases and controls. There may also be healthy bias at play; participants of the present study, both cases and controls, were required to attend the study centre for an assessment which may not be achievable for those less well. This bias was recently demonstrated when comparing participants in a 2-year exercise randomised controlled trial for falls prevention against non-participants; participants were found to be physically and mentally healthier with greater functional capacity than non-participants [36]. We were not able to determine if there were differences between those included in the study and non-participants, thus, a selection bias may be present. Finally, these results may not be generalizable to men with bipolar disorder.

In conclusion, women with bipolar disorder are at excess risk of falls. The detrimental outcomes associated with falls, in particular fractures in injurious falls, and fear and anxiety can lead to restriction in daily activities and decreased quality of life. Thus, this relationship warrants further investigation and clinical attention. Exploring the diversity of possible underlying mechanisms contributing to the increased risk of falls, could inform falls prevention programs and enable clinicians to identify those at highest risk.

## Data Availability

The datasets used and/or analysed during the current study are available from the corresponding author on reasonable request.

## Abbreviations

BMI: Body mass index
BP: Blood pressure
BSD: Barwon Statistical Division
CI: Confidence interval
GEE: Generalised Estimating Equations
GOS: Geelong Osteoporosis Study
IRSAD: Index of relative socioeconomic advantage and disadvantage
NOS: Not otherwise specified
OR: Odds ratio
SEIFA: Socio-economic indexes for areas
SES: Socioeconomic status
SCID-I/NP: Structured Clinical Interview for DSM-IV-TR Research Version, Non-patient edition
